# An IMD-like pathway mediates both endosymbiont control and host immunity in the cereal weevil *Sitophilus* spp.

**DOI:** 10.1186/s40168-017-0397-9

**Published:** 2018-01-08

**Authors:** Justin Maire, Carole Vincent-Monégat, Florent Masson, Anna Zaidman-Rémy, Abdelaziz Heddi

**Affiliations:** 1 Univ Lyon, INSA-Lyon, INRA, BF2i, UMR0203, F-69621 Villeurbanne, France; 2 Present address: Global Health Institute, School of Life Sciences, Ecole Polytechnique Fédérale de Lausanne (EPFL), Station 19, 1015 Lausanne, Switzerland

**Keywords:** Endosymbiosis, Bacteriocyte, Antimicrobial peptide, *Sitophilus*, Innate immunity, Compartmentalization

## Abstract

Many insects developing on nutritionally unbalanced diets have evolved symbiotic associations with vertically transmitted intracellular bacteria (endosymbionts) that provide them with metabolic components, thereby improving the host’s abilities to thrive on such poor ecological niches. While host-endosymbiont coevolutionary constraints are known to entail massive genomic changes in the microbial partner, host’s genomic evolution remains elusive, particularly with regard to the immune system. In the cereal weevil *Sitophilus* spp., which houses *Sodalis pierantonius*, endosymbionts are secluded in specialized host cells, the bacteriocytes that group together as an organ, the bacteriome. We previously reported that at standard conditions, the bacteriome highly expresses the *coleoptericin A* (*colA*) antimicrobial peptide (AMP), which was shown to prevent endosymbiont escape from the bacteriocytes. However, following the insect systemic infection by pathogens, the bacteriome upregulates a cocktail of AMP encoding genes, including *colA*. The regulations that allow these contrasted immune responses remain unknown. In this short report, we provide evidence that an IMD-like pathway is conserved in two sibling species of cereal weevils, *Sitophilus oryzae* and *Sitophilus zeamais*. RNA interference (RNAi) experiments showed that *imd* and *relish* genes are essential for (i) *colA* expression in the bacteriome under standard conditions, (ii) AMP up-regulation in the bacteriome following a systemic immune challenge, and (iii) AMP systemic induction following an immune challenge. Histological analyses also showed that *relish* inhibition by RNAi resulted in endosymbiont escape from the bacteriome, strengthening the involvement of an IMD-like pathway in endosymbiont control. We conclude that *Sitophilus*’ IMD-like pathway mediates both the bacteriome immune program involved in endosymbiont seclusion within the bacteriocytes and the systemic and local immune responses to exogenous challenges. This work provides a striking example of how a conserved immune pathway, initially described as essential in pathogen clearance, also functions in the control of mutualistic associations.

**Electronic supplementary material:**

The online version of this article (10.1186/s40168-017-0397-9) contains supplementary material, which is available to authorized users.

## Main text

Host-symbiont associations are widespread in nature and exhibit a variety of interactions ranging from parasitism to mutualism. Insects living on nutritionally unbalanced diets are prone to establish long-term mutualistic relationships with vertically transmitted intracellular bacteria (endosymbionts) that complement their diet, improve their metabolism and reproduction, and impact many host adaptive traits, including immunity and defense against pathogens [1–7]. While the metabolic, ecological and evolutionary features of these interactions have been well described [8–10], the mechanisms allowing the persistence of such associations remain largely unexplored. Beneficial bacteria are essential for the association’s survival but represent a constant immune challenge for the host. Insect immunity must preserve endosymbionts and control their load and location while being able to cope with potential environmental infections by microbial intruders. This dilemma is more puzzling considering that both pathogenic and beneficial interactors display conserved immune elicitors, the microbe-associated molecular patterns (MAMPs; e.g., peptidoglycan (PGN)), that are sensed by innate immune receptors. In long-lasting endosymbiotic associations, host-symbiont coevolution is accompanied by a bacterial genomic erosion that generally results in the loss of bacterial genes that are redundant or harmful for the association [8, 9, 11]. Among these, genes encoding enzymes involved in MAMP synthesis can be lost, such as in *Buchnera aphidicola*, the pea aphid’s primary endosymbiont [12]. This feature was suggested to enable endosymbionts to avoid recognition by the host’s immune system. On the host’s side, many insects have selected a compartmentalization strategy that consists in secluding endosymbionts within specialized host cells, the bacteriocytes (which form a bacteriome organ in some insect species), limiting thereby their direct contact with the host systemic immune response [5, 10, 13–16]. Compartmentalization plays several functions, including centralization of host-symbiont metabolic exchanges [17, 18], control of endosymbiont load and location [19–23], and endosymbiont preservation from exogenous pathogens [24]. Similar symbiont compartmentalizations can be found in amphibians [25], plants [26, 27], cnidarians [28], and mollusks [29, 30]. For instance, in the squid *Euprymna scolopes*, symbiont compartmentalization in a light organ is essential for efficient light production and symbiont population control [31–33].

Among insects, the cereal weevil *Sitophilus* spp. mutualistic association with the Gram-negative bacterium *Sodalis pierantonius* [13, 34, 35] is relevant to address endosymbiont-host immune interactions. *S. pierantonius* has been acquired recently by cereal weevils (less than 30,000 years ago) [36], likely following the replacement of *Candidatus* Nardonella, the ancestor endosymbiont of the Dryophthoridae family [37, 38]. The genome of *S. pierantonius* has not experienced the size shrinkage usually observed in endosymbionts, and it notably retains genes encoding enzymes involved in MAMP synthesis, including PGN [34]. Injection of *S. pierantonius* in the weevil’s hemolymph elicits a systemic immune response, attested by a high induction of antimicrobial peptide (AMP)-encoding genes [39]. Under standard conditions (i.e., in the absence of infection with free-living bacteria), we have previously shown that the weevil’s bacteriome displays a specific immune program, the so-called bacteriome “internal response” [40], which is essential for *S. pierantonius* seclusion. Despite the massive endosymbiont load, most AMP-encoding genes are weakly expressed in the bacteriome, with the notable exception of the *coleoptericin A* (*colA*) gene [17, 20, 39, 41, 42]. ColA peptide was shown to enter the bacterial cytosol and to interact with several bacterial proteins, including the chaperonin GroEL, resulting in the inhibition of bacterial cytokinesis and the formation of gigantic, filamentous, and polyploid endosymbionts [20, 43]. In vivo functional analyses revealed that ColA functions as a “molecular guard” that prevents endosymbiont escape from the bacteriome [20]. The bacteriome is also able to mount a local immune response upon systemic infection by free-living microbes, called the bacteriome “external response” [40]. Bacteriomes of challenged insects upregulate the expression of a cocktail of AMP-encoding genes, including *colA* but also *coleoptericin B* (*colB*) and *sarcotoxin* [24]. The molecular basis of these contrasted immune responses remains elusive, as well as more generally the immune pathways regulating the weevil immune response to Gram-negative bacterial infections.

Knowledge on insect humoral immunity is largely acquired from the *Drosophila* model, in which Gram-negative bacteria are detected by immune receptors that recognize their DAP-type PGN, or derived monomeric fragments, including the tracheal cytotoxin (TCT) [44, 45]. PGN or TCT recognition triggers the activation of a signaling cascade, the immune deficiency (IMD) pathway, that leads to the activation and nuclear translocation of the NF-κB transcription factor Relish, which in turn upregulates AMP expression [46]. Since its discovery in *Drosophila*, the IMD pathway was described in many other species [47–50], attesting its high conservation across insect groups.

**Fig. 1:**
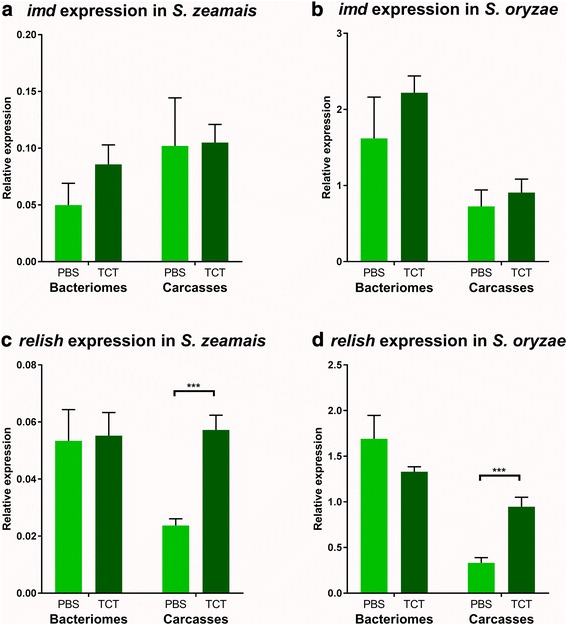
*imd* and *relish* expression patterns. *imd* and *relish* expression was measured by RT-qPCR in bacteriomes and carcasses. Tissues were dissected 6 h following either PBS or TCT injection, 6 days after *gfp* dsRNA injection. Gene expression was normalized by the geometric mean of two housekeeping gene expressions, *rpl29* and *mdh*. **a** *imd* expression in *S. zeamais*. **b** *imd* expression in *S. oryzae*. **c** *relish* expression in *S. zeamais*. **d** *relish* expression in *S. oryzae*. The mean and standard error for five independent replicates are represented. Asterisks indicate a significant difference between two conditions based on a Welch’s *t* test (*p < 0.05; **p < 0.01; ***p < 0.001)

In this study, we first sought to determine whether the IMD pathway is conserved in the cereal weevil. We looked in two weevil sibling species, *Sitophilus zeamais* and *Sitophilus oryzae*, at both the gene conservation and the expression patterns of *imd* and *relish*, which are the two key genes of the IMD pathway in *Drosophila*. Both *imd* and *relish* gene transcripts had previously been found in the rice weevil *S. oryzae* transcriptome [24, 42], and partial sequences were subsequently identified in *S. zeamais* based on *S. oryzae* sequences. We next examined *imd* and *relish* expression patterns in the bacteriome and in the rest of the larva (larval body after bacteriome dissection, hereafter referred to as “the carcass”), under standard conditions and after an immune challenge. To avoid any potential interference of living bacteria that can actively modulate host immunity, we chose to inject larvae with purified *Escherichia coli* TCT. Injection with sterile phosphate buffer saline (PBS) is the control condition in this study and represents the basal expression of all genes studied. Systemic TCT injection in *S. zeamais* larvae efficiently induced the expression of three AMPs used as reporter genes for immune activation (*colA*, *colB*, and *sarcotoxin*), at 6 h post-injection, as compared to larvae injected with PBS (Additional file 1). This immune response is similar to what has been reported in *Drosophila* [44] and to what is observed when injecting various Gram-negative bacteria in the weevil’s hemolymph [24, 39, 51]. In both weevil species, *imd* and *relish* genes were expressed in the bacteriome and the carcass (Fig. 1). *Imd* expression did not show any difference between PBS- and TCT-injected larvae in both the bacteriome and the carcass (Fig. 1a, b). Following insect challenge with TCT, *relish* expression level remained stable in the bacteriome but it highly increased in the larval carcass (Fig. 1c, d). The existence of the genes and transcripts associated with these two components in the bacteriome and the carcass suggests that an IMD-like pathway is conserved in the cereal weevil, similarly to *Drosophila* [52], the coleopteran *Tribolium castaneum* [47], and the dipteran *Glossina morsitans* [48]. It is noteworthy that the IMD pathway has been lost in the pea aphid *Acyrtosiphon pisum*, along with many immune genes including peptidoglycan recognition proteins and known AMPs. This immune gene degeneration was speculated to be an adaptation of aphids to their symbiotic association [53, 54].

**Fig. 2:**
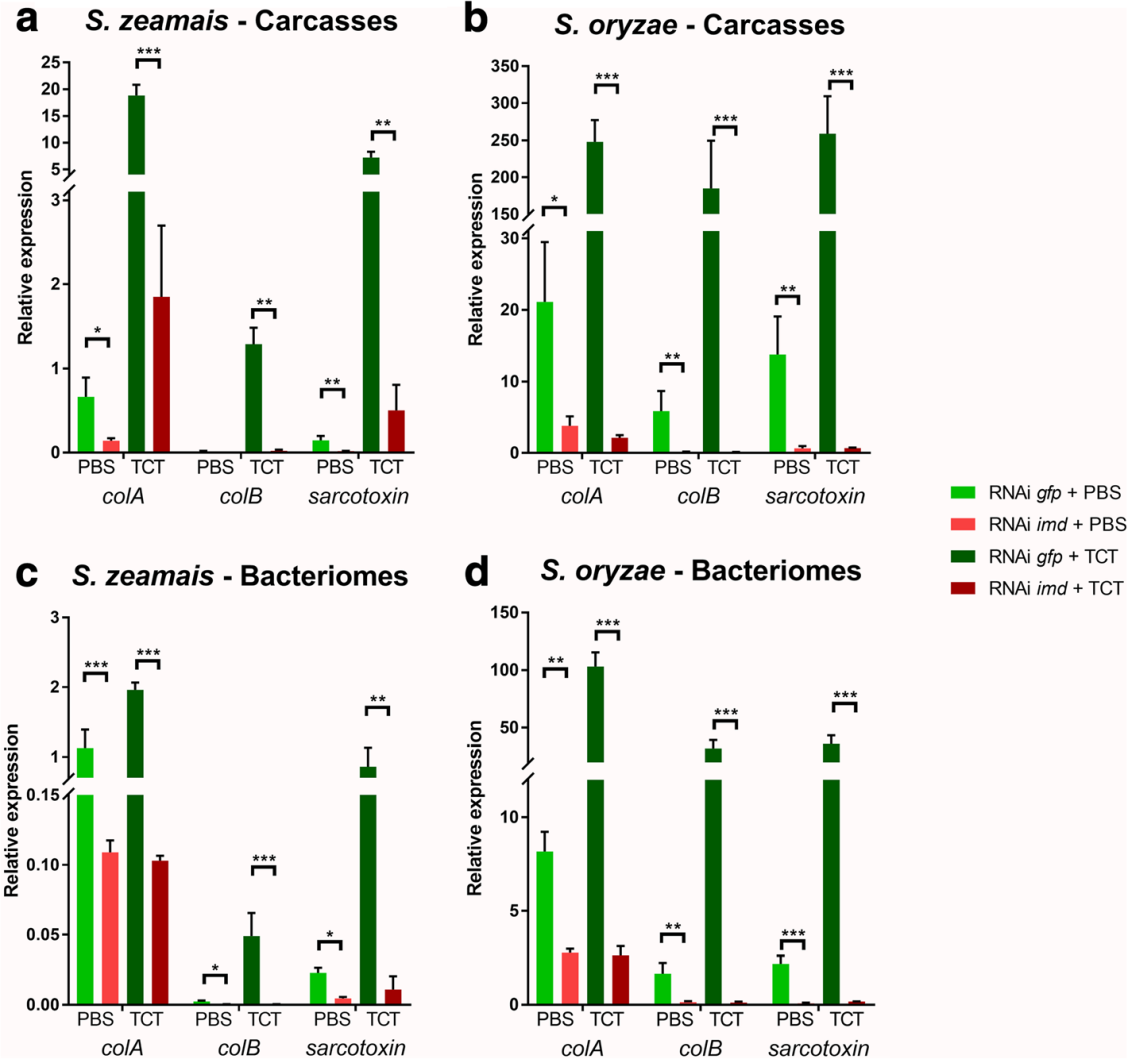
AMP expression is IMD-dependent in *S. zeamais* and *S. oryzae*. *colA*, *colB*, and *sarcotoxin* expressions were measured by RT-qPCR 6 h after either PBS or TCT injections, following *gfp* or *imd* extinction. Gene expression was normalized by the geometric mean of two housekeeping gene expressions, *rpl29* and *mdh*. **a** AMP expression in *S. zeamais*’ carcasses. **b** AMP expression in *S. oryzae*’s carcasses. **c** AMP expression in *S. zeamais*’ dissected bacteriomes. **d** AMP expression in *S. oryzae*’s dissected bacteriomes. The mean and standard error for five independent replicates are represented. Asterisks indicate a significant difference between two conditions based on a Welch’s *t* test (*p < 0.05; **p < 0.01; ***p < 0.001)

To assess whether and how this IMD-like pathway is involved in the weevil’s immune responses, we first used RNA interference (RNAi) against *imd* transcripts in both species [55]. The steady-state levels of *imd* transcripts drastically decreased following larval injection with dsRNA in both the bacteriome and the carcass and in all conditions (Additional file 2). As expected, TCT injection induced AMP expression in the carcass as compared to PBS-injected larvae (Fig. 2a, b). Importantly, *imd* RNAi led in both weevil species to a significant inhibition of the basal and induced AMP expression in the carcass, after PBS and TCT injection, respectively. IMD is thus required for systemic AMP induction following the injection of Gram-negative bacterial PGN in *Sitophilus*, similarly to what has been reported in other arthropods [52, 56, 57]. Interestingly, we showed that the steady-state levels of all AMP transcripts were strongly downregulated in the bacteriome, following *imd* inhibition by RNAi in PBS-injected larvae of both *S. zeamais* and *S. oryzae* (Fig. 2c, d). These findings indicate that, in the absence of infection with exogenous bacteria, all three AMPs, whether strongly (*colA*) or weakly expressed (*colB* and *sarcotoxin*), are under tight regulation of IMD in the bacteriome. Similarly to larval challenge with the Gram-negative bacterium *Dickeya dadantii* [24], injection of larvae with TCT resulted in the upregulation of all three AMPs in the bacteriome, when compared to PBS-injected larvae. The induction of the bacteriome “external response” was strongly reduced in larvae treated with *imd* RNAi, in both species (Fig. 2c, d). Therefore, IMD not only activates the systemic immune response to TCT, but it also mediates the bacteriome “internal” and “external” responses. Despite different expression patterns, systemic and bacteriome local AMP expressions seem to be regulated by the same pathway. It was reported in *Drosophila* that AMP expression and regulation can be tissue-dependent. For instance, the *drosomycin* gene expression was shown to be Toll-dependent in the fat body [58], IMD-dependent in the epithelia [59], while its constitutive expression in the salivary glands and the gut has been attributed to the homeobox gene *caudal* [60, 61]. It will be interesting to figure out whether the regulation of AMP expression by IMD applies to all AMPs and all tissues in the weevil, or if other pathways are involved.

**Fig. 3:**
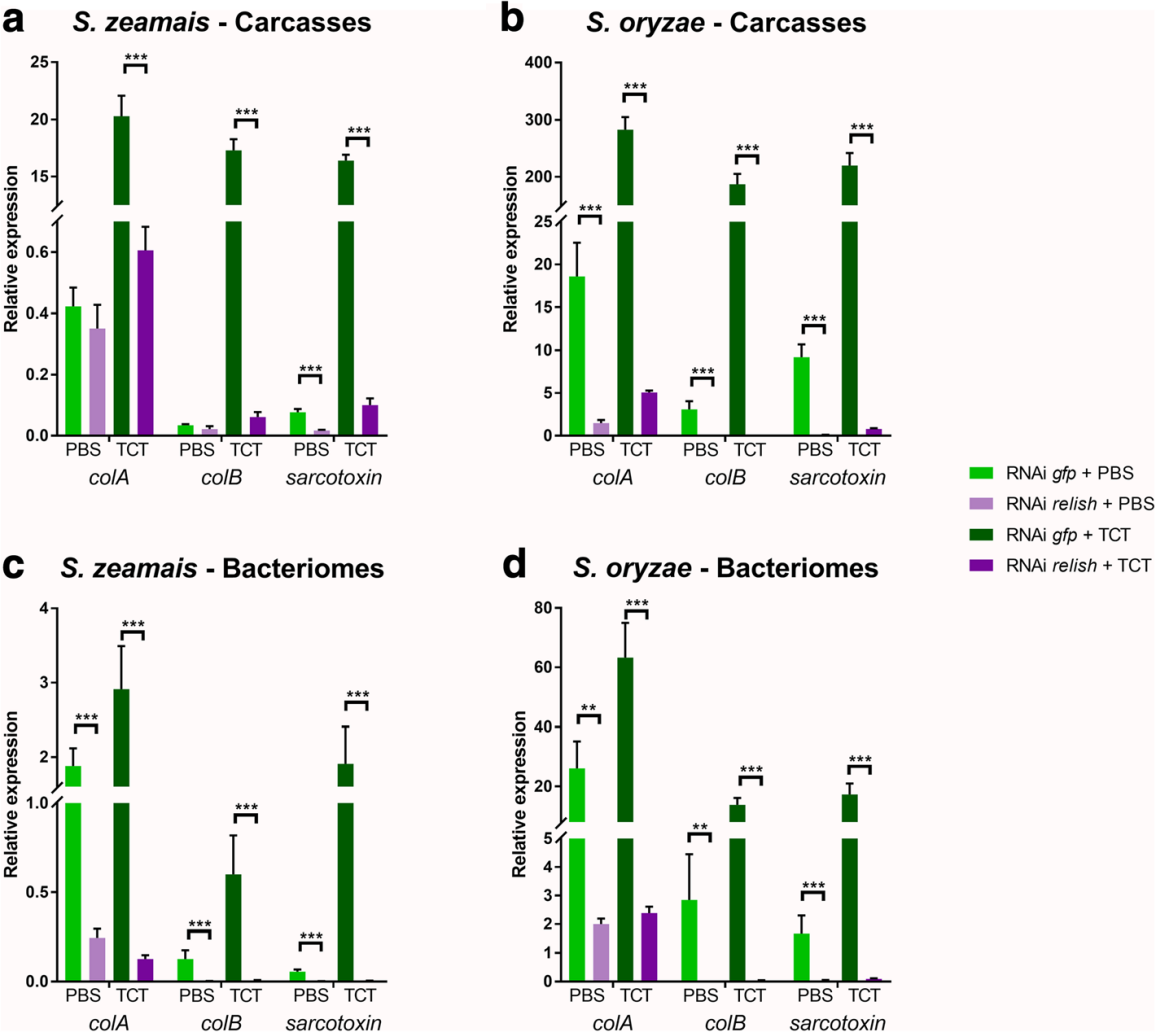
AMP expression is Relish-dependent in *S. zeamais* and *S. oryzae*. *colA*, *colB*, and *sarcotoxin* expressions were measured by RT-qPCR 6 h after either PBS or TCT injections, following *gfp* or *relish* extinction. Gene expression was normalized by the geometric mean of two housekeeping gene expressions, *rpl29* and *mdh*. **a** AMP expression in *S. zeamais*’ carcasses. **b** AMP expression in *S. oryzae*’s carcasses. **c** AMP expression in *S. zeamais*’ dissected bacteriomes. **d** AMP expression in *S. oryzae*’s dissected bacteriomes. The mean and standard error for five independent replicates are represented. Asterisks indicate a significant difference between two conditions based on a Welch’s *t* test (*p < 0.05; **p < 0.01; ***p < 0.001)

Because IMD appears to control two distinct responses in the weevil bacteriome, i.e. the “internal” symbiosis-related program and the “external” immune response to TCT injection, we wondered whether this pathway could split into two distinct transduction signals downstream of IMD, leading to the differential recruitment of transcription factors that could account for the two responses. To test this hypothesis, we inhibited *relish* expression by RNAi (Additional file 2) and monitored AMP expression in PBS-injected and TCT-injected larvae. Remarkably, AMP transcript profiles were highly similar when compared with those following *imd* inhibition, in all conditions and in both weevil species (Fig. 3). These data indicate that Relish is required for the basal expression of most AMPs in the carcass of PBS-injected larvae, as well as for the systemic AMP upregulation in response to TCT injection (Fig. 3a, b). Moreover, Relish was required for the bacteriome “internal” response program in PBS-injected larvae, as *colA* expression was significantly decreased in larvae subjected to *relish* RNAi (Fig. 3c, d). Finally, Relish was also required for AMP induction in the bacteriome after TCT injection (Fig. 3c, d). These data indicate that the bacteriome “internal” and “external” immune responses, as well as the systemic immune response, are all Relish-dependent in both *S. zemais* and *S. oryzae*. Nonetheless, additional transcription factors might be at play. A qualitatively or quantitatively different set of transcription factor binding sites upstream of the respective AMP encoding genes could result in their differential expression. For instance, *Drosophila*’s Pickle specifically inhibits Relish homodimers, therefore only impacting the expression of AMP genes containing two Relish binding sites [62]. *Sitophilus* genome will be soon available, which will open a field of investigation on the AMP-encoding gene promoting sequences and the corresponding transcription factors.

**Fig. 4:**
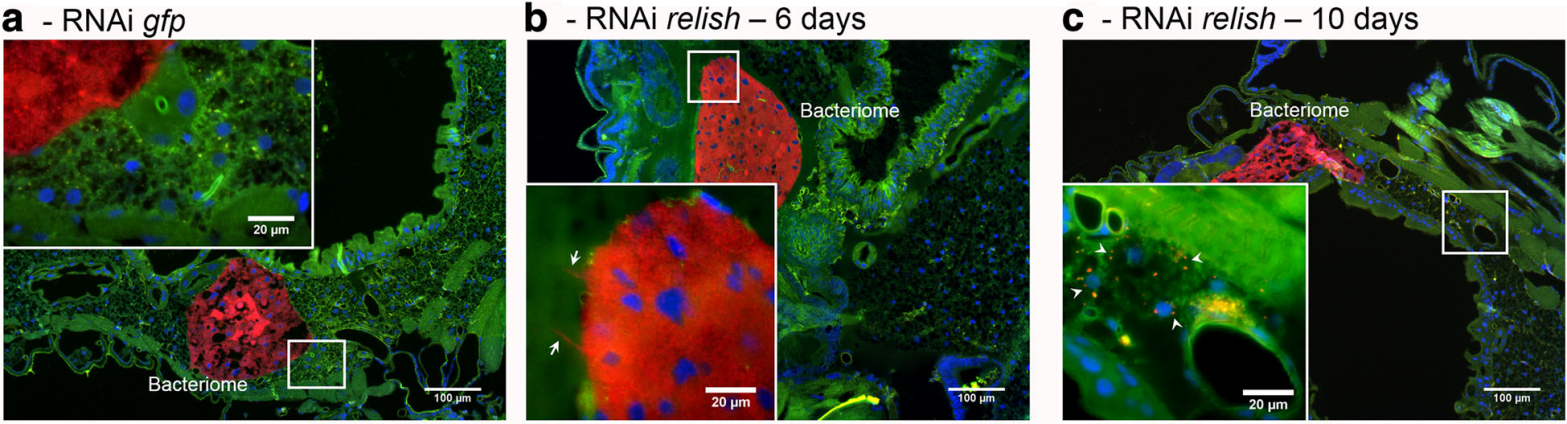
*S. pierantonius* localization by FISH following *relish* inhibition in *S. zeamais* larvae. Red, *S. pierantonius*; green, autofluorescence; blue, DAPI. **a** Six days following *gfp* dsRNA injection. **b** Six days following *relish* dsRNA injection. Arrows point at endosymbionts exiting from the bacteriome. **c** Ten days following *relish* dsRNA injection. Arrowheads point at endosymbionts present in the fat body

Finally, FISH experiments were conducted on *S. zeamais* larvae after *relish* inhibition, in order to specifically localize *S. pierantonius* and to detect any potential loss of endosymbiont control. Symbionts were seen “leaking” from the bacteriome 6 days after *relish* dsRNA injection (Fig. 4b), and many bacteria were seen in the larval fat body 10 days after *relish* dsRNA injection (Fig. 4c). This phenotype is similar to what was previously observed following *colA* inhibition [20] and confirms that, by regulating *colA* expression, this IMD-like pathway is directly involved in symbiosis compartmentalization.

Taken together, these findings indicate that *colA* expression in the bacteriome is under the control of an IMD-like pathway, involving at least IMD and Relish. Even though several components of the IMD pathway have been suggested to be involved in symbiotic interactions [63–65], this is the first demonstration that an IMD-like pathway is involved in endosymbiont control. Nevertheless, because *colA* expression in the bacteriome was not completely inhibited under either *imd* or *relish* RNAi conditions, we cannot rule out the implication of another pathway in *colA* regulation, although this incomplete downregulation could also be the result of the incomplete RNAi-driven knock-down of *imd* and *relish* expression. Strikingly, the “internal response” and the “external response” are both under the same IMD/Relish-dependent regulation. What remains intriguing is that *colA*, despite its strong expression in the bacteriome under basal conditions, is under the same regulation as other AMPs, whose genes are weakly expressed under basal conditions. The IMD dependency of *colA* expression under basal conditions argues in favor of a symbiont-induced basal expression rather than a symbiont-independent expression. This hypothesis is reinforced by previous results showing that *colA* expression matches the drastic symbiont density changes along the insect lifecycle [40, 66]. Sensing the endosymbiont load would allow the host to modulate the internal response accordingly. As endosymbionts are exclusively intracellular, they could be sensed by an intracellular receptor, similar to the *Drosophila* PGRP-LE [67], whereas exogenous pathogens could be recognized by an extracellular or transmembrane receptor, similar to the *Drosophila* PGRP-LC [68, 69]. Such a dichotomous mechanism would allow distinguishing endosymbionts and pathogens at the recognition step and could differentially impact AMP expression while activating the same IMD pathway.

The data presented here strongly suggest that the specific and fine-tuned regulation of the bacteriome immune responses involves more complex mechanisms beyond a simple dichotomy in the implicated signal transduction pathways. Deciphering such complex regulations is likely to require the study of potential epigenetic markers, such as chromatin and histone modifications, that are increasingly shown to be involved in symbiotic interactions [70]. This will include addressing the possibility of non-coding RNA implication in the host immune regulations and their potential manipulation by the endosymbiont. Recent studies in insects have shown that several microRNAs specifically target and regulate immune genes, including AMPs [71, 72], and can mediate host-symbiont interactions as shown in the *Aedes aegypti-Wolbachia* association [73].

## Conclusion

We demonstrated that (i) the weevil’s immune system expresses a functional IMD-like pathway; (ii) this IMD-like pathway regulates the systemic immune response to Gram-negative bacterial MAMPs; and (iii) both the “external” and “internal” bacteriome immune responses are IMD/Relish-dependent. Interestingly, this study uncovers that a highly conserved immune pathway is involved in both the weevil immune response to microbe intruders and in the regulation of the symbiosis-related specific program. This is the first report showing that an insect endosymbiosis compartmentalization strategy relies on an IMD-like immune pathway. The finding that IMD and Relish regulate both the internal and external responses of the weevil bacteriome is the first milestone on the long path to the full understanding of immune adaptations to endosymbiosis.

## Methods

### Biological material and sample preparation

*Sitophilus* weevils were reared on wheat grains at 27.5 °C and at 70% relative humidity [74]. The Lagoa (*S. zeamais*) and Bouriz (*S. oryzae*) strains were chosen in this work because they are devoid of any facultative symbionts, including *Wolbachia*.

Insect bacteriomes and carcasses were dissected in buffer A (25 nM KCl, 10 nM MgCl2, 250 nM Sucrose, 35 nM Tris/HCl, pH = 7.5). For RNA extraction, at least five organs or whole organisms per condition were pooled and stored at − 80 °C, and each sampling was independently repeated five times.

### Identification of genes of interest

The AMP-encoding genes *colA, colB*, and *sarcotoxin* were identified in *S. zeamais* and *S. oryzae* from previous studies [39, 42], as well as *imd* and *relish* in *S. oryzae* [24, 42]. *Imd* and *relish* in *S. zeamais* were identified based on the homology with *S. oryzae* sequences and unpublished genomic and transcriptomic data. The respective sequences can be found under the following accession numbers: MF952871 and MF952872.

### TCT injections

Injections were made on fourth instar larvae challenged with a 0.2 mM TCT solution purified from *E. coli* as previously described [44]. Fifty-five nanoliters was injected into the hemolymph using a Nanoject II (Drummond). Sterile PBS was used as a negative control. Larvae were then incubated in wheat flour at 27.5 °C and at 70% relative humidity during 6 h.

### dsRNA synthesis and injection

dsRNA was prepared as described previously [55]. Primers used for T7 DNA fragments are listed in Additional file 3 and were designed to amplify a fragment from 200 to 250 pb. Fragments were amplified with a Taq’ozyme kit (Ozyme) and purified with a GenElute PCR Clean-up kit (Sigma-Aldrich), following the manufacturer’s instructions. These fragments were used as templates for in vitro dsRNA synthesis, using a MEGAscript RNAi Kit (Ambion). After synthesis, the dsRNA was precipitated overnight at − 80 °C with 0.3 M sodium acetate, 1.5 μg glycogen, and two volumes of 100% EtOH and resuspended in water to a final concentration of 2.7 μg/μl. The purity and the integrity were determined with a Nanodrop® spectrophotometer (Thermo Scientific) and by agarose gel electrophoresis. The dsRNA was kept at − 20 °C prior to injection within the following 7 days. Fifty nanograms of dsRNA was injected into the hemolymph of third instar larvae with a Nanoject II (Drummond). They were then kept on wheat flour for 6 to 10 days, at 27.5 °C and at 70% relative humidity.

### Total RNA extraction and reverse transcription

Total RNA from whole larvae and carcasses was extracted with TRIzol reagent (Invitrogen) following the manufacturer’s instructions. RNA was incubated with 1 U/μg of RQ1 RNase-free DNase (Promega) for 30 min at 37 °C and purified using Nucleospin RNAClean-up (Macherey-Nagel). Total RNA from bacteriomes was extracted and purified using RNAqueous Micro (Ambion), which allows for a better RNA yield from small tissue samples. After purification, the RNA concentration was measured with a Nanodrop® spectrophotometer (Thermo Scientific), and RNA quality was checked using agarose gel electrophoresis. Reverse transcription into the first strand cDNA was carried out using the iScript™ cDNA Synthesis Kit (Bio-Rad).

### Real-time RT-qPCR transcript quantification

The transcript quantification was performed with a CFX Connect Real-Time detection system (Bio-Rad) using the LightCycler Fast Start DNA Master SYBR Green I kit (Roche Diagnostics). Data were calculated using the ratio of the target cDNA concentration to the geometric mean of two normalizing gene concentrations: ribosomal protein L29 (*rpL29*) and malate dehydrogenase (*mdh*). Primers were designed to amplify fragments of approximately 150–200 bp. A complete list of the primers can be found in Additional file 3.

The PCR reactions were carried out in Hard-Sell 96-well PCR plates (Bio-Rad) in a final volume of 10 μl, containing 2.5 μl of cDNA samples (diluted fivefold) with 0.5 μl of 10 mM of each primer, 1.5 μl H_2_O, and 5 μl of Sybr Mastermix. After 5 min at 95 °C, the cycling conditions were as follows: 45 cycles at 95 °C for 10 s, 56 °C for 20 s, and 72 °C for 30 s. For product identification, a melting curve was constructed at the end of each PCR by heating for 30 s at 66 °C and then increasing the temperature up to 95 °C with increment rates of 0.11 °C/s. Reactions were terminated by cooling at 40 °C for 30 s. For each individual sample, the crossing point and the concentration of the gene transcripts were determined.

### Statistical analyses

Transcriptomic data were analyzed by pairwise comparisons using a Welch *t* test on the log-transformed gene expression data. The effect of a factor was considered to be significant with a *p* value < 0.05. All analyses and graphical figures were made using R software v3.1.1 [75], as well as GraphPad Prism v7 for Windows, GraphPad Software, La Jolla California USA, www.graphpad.com. Graphical figures represent the mean of all replicates for each point. Error bars represent the standard error calculated as *σ*/√*n*, where *σ* is the standard deviation and *n* is the number of replicates.

### Immunohistochemistry

#### Sample preparation for histology

Samples were fixed in PFA 4%. After 1 week at 4 °C, the fixative was replaced by several washings with PBS before embedding the tissue in 1.3% agar. Subsequently, the samples were dehydrated through a graded ethanol (EtOH) series and transferred to butanol-1, at 4 °C, overnight. Samples in agar were then embedded in melted Paraplast. Tissue sections (3-μm thick) were cut using an LKB Historange microtome. Sections were placed on poly-lysine-coated slides, dried overnight in a 37 °C oven, and stored at 4 °C prior to further treatments.

#### *S. pierantonius* localization by fluorescence in situ hybridization

After methylcyclohexane dewaxing, sections were covered with a drop of 70% acetic acid. Deproteinization of slides was performed in hydrochloric acid 0.01 N with pepsin 0.1 mg/ml for 10 min at 37 °C. The sections were then prehybridized, hybridized with a *S. pierantonius*-specific 5′-end TAMRA-labeled oligo-probe targeting 16S RNA (TAMRA-ACC-CCC-CTC-TAC-GAG-AC-3′, 10 μg/mL), washed, and then mounted in PermaFluor Mounting Fluid (ThermoScientific) containing 3 μg/ml of 4′,6-diamidino-2-phenylindole (DAPI), as previously described [15].

Images were acquired with an epifluorescence microscope (Olympus IX81 equipped with a HQ535/50 filter for green signal, D470/40 for blue signal, and HQ610/75 for red signal) and captured using an F-ViewII camera and the cellSens software (Olympus). Images were treated and analyzed using ImageJ (release 1.47v).

## Additional files

Additional file 1: AMP expression following TCT injection in *S. zeamais*. *colA*, *colB*, and *sarcotoxin* expressions were measured by RT-qPCR in whole larvae 6 h following either PBS or TCT injection, 6 days after *gfp* dsRNA injection. Gene expression was normalized by the geometric mean of two housekeeping gene expressions, *rpl29* and *mdh*. The mean and standard error for five independent replicates are represented. Asterisks indicate a significant difference between two conditions based on a Welch’s *t* test (*p < 0.05; **p < 0.01; ***p < 0.001). (TIFF 441 kb)

Additional file 2:*imd* and *relish* inhibition by RNAi in *S. zeamais* (a) and *S. oryzae* (b). *imd* and *relish* expressions were measured by RT-qPCR in bacteriomes and carcasses. Tissues were dissected 6 h following either PBS or TCT injection, 6 days after *gfp*, *imd*, or *relish* dsRNA injection. Gene expression was normalized by the geometric mean of two housekeeping gene expressions, *rpl29* and *mdh*. Raw data (mean ± SD) as well as inhibition percentages are provided. Percentages were calculated as follows:$\%=\left(1-\frac{imd\ \mathrm{RNAi}\  \mathrm{or}\  relish\ \mathrm{RNAi}\ }{gfp\ \mathrm{RNAi}\ }\right)\times 100$; using the mean of five independent measurements for each value. Asterisks indicate a significant difference between a condition and its corresponding *gfp* RNAi control based on a Welch’s *t* test (*p < 0.05; **p < 0.01; ***p < 0.001). (XLSX 11 kb)

Additional file 3: List of primers used for dsRNA synthesis and RT-qPCR. (XLSX 12 kb)

## Abbreviations

AMP: Antimicrobial peptide
Col: Coleoptericin
DAP: Diaminopimelic acid
FISH: Fluorescent in situ Hybridization
IMD: Immune deficiency
MAMP: Microbial-associated molecular pattern
PBS: Phosphate buffer saline
PGN: Peptidoglycan
RNAi: RNA interference
TCT: Tracheal cytotoxin
