# Supplementary figures and images for: An IMD-like pathway mediates both endosymbiont control and host immunity in the cereal weevil Sitophilus spp
Source: Microbiome. 2018 Jan 8;6:6. doi: 10.1186/s40168-017-0397-9 (PMC5759881; doi:10.1186/s40168-017-0397-9)

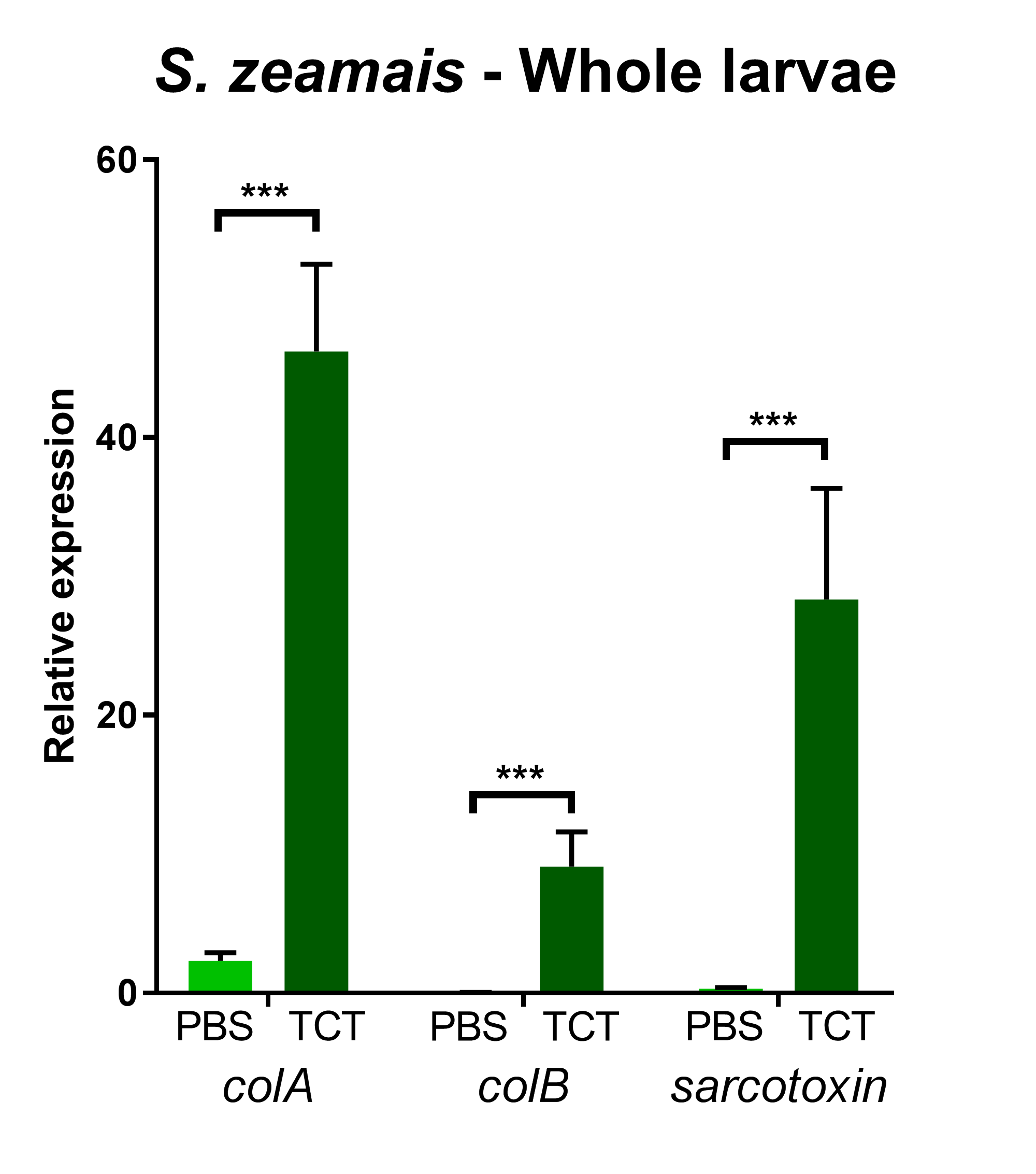

Supplement: Supplementary file 1 — AMP expression following TCT injection in S. zeamais. colA, colB, and sarcotoxin expressions were measured by RT-qPCR in whole larvae 6 h following either PBS or TCT injection, 6 days after gfp dsRNA injection. Gene expression was normalized by the geometric mean of two housekeeping gene expressions, rpl29 and mdh. The mean and standard error for five independent replicates are represented. Asterisks indicate a significant difference between two conditions based on a Welch’s t test (*p < 0.05; **p < 0.01; ***p < 0.001). (TIFF 441 kb) [file 40168_2017_397_MOESM1_ESM.tif]
